# Plasma biomarkers for diagnosis and differentiation and their cognitive correlations in patients with Alzheimer’s disease

**DOI:** 10.1093/braincomms/fcaf094

**Published:** 2025-02-25

**Authors:** Wenhao Sun, Shuwei Ye, Yu Wang, Huifeng Chen, Ping Che, Jingshan Chen, Nan Zhang

**Affiliations:** Department of Neurology, Tianjin Neurological Institute, Tianjin Medical University General Hospital, Tianjin 300052, China; Department of Neurology, Tianjin Neurological Institute, Tianjin Medical University General Hospital, Tianjin 300052, China; Department of Neurology, People's Hospital of Qingxian, Hebei 062650, China; Department of Neurology, Tianjin Neurological Institute, Tianjin Medical University General Hospital, Tianjin 300052, China; Department of Neurology, Tianjin Medical University General Hospital Airport Site, Tianjin 300300, China; Department of Neurology, Tianjin Neurological Institute, Tianjin Medical University General Hospital, Tianjin 300052, China; Department of Neurology, Tianjin Neurological Institute, Tianjin Medical University General Hospital, Tianjin 300052, China; Department of Neurology, Tianjin Neurological Institute, Tianjin Medical University General Hospital, Tianjin 300052, China

**Keywords:** plasma biomarker, Alzheimer’s disease, amyloid β, P-tau181, glial fibrillar acidic protein

## Abstract

Increasing evidence has shown the potential value of plasma biomarkers in Alzheimer’s disease diagnosis. This study aimed to determine the diagnostic and differential values of emerging plasma biomarkers for different types of dementia in a Chinese population and to explore their cognitive correlations. One hundred twenty patients with dementia, including 51 Alzheimer’s disease patients, 54 subcortical ischaemic vascular dementia (SIVD) patients and 15 frontotemporal lobar degeneration (FTLD) patients were recruited alongside 27 cognitively unimpaired (CU) control subjects. Global and domain-specific cognition was assessed in all participants by a battery of neuropsychological tests. Plasma amyloid-beta (Αβ)42, Aβ40 and total tau (in CU controls and Alzheimer’s disease patients) and phosphorylated tau at threonine-181 (*P*-tau181), neurofilament light (NfL) and glial fibrillar acidic protein (GFAP) levels (in all participants) were measured using the single-molecule array platform. The levels of all biomarkers differed between Alzheimer’s disease patients and CU controls, with *P*-tau181 and GFAP levels and the Aβ42/P-tau181 ratio best differentiating the two groups [area under the curve (AUC) = 0.966, 0.932 and 0.927, respectively]. P-tau181 and GFAP levels were greater in the Alzheimer’s disease group than in the other two patient groups and showed the best performance in distinguishing Alzheimer’s disease patients from SIVD (AUC = 0.922) and FTLD patients (AUC = 0.894), respectively. Moreover, compared with that in the CU group, the GFAP level was elevated in the SIVD group, and the NfL level was elevated in all patient groups. Compared with other single biomarkers, the plasma Aβ42/P-tau181 ratio correlated with broader cognitive domains, including global cognition [Mini-Mental Status Examination (MMSE), *r* = 0.314, *P* = 0.027; Montreal Cognitive Assessment (MoCA), *r* = 0.313, *P* = 0.043], memory (*r* = 0.339, *P* = 0.016), language (*r* = 0.333, *P* = 0.020), attention and information processing speed (*r* = 0.369, *P* = 0.008), executive function (*r* = 0.305, *P* = 0.031) and visuospatial function memory (*r* = 0.453, *P* = 0.001). P-tau181 was an optimal plasma biomarker for identifying Alzheimer’s disease patients and differentiating Alzheimer’s disease patients from SIVD and FTLD patients. Moreover, the GFAP level and the Aβ42/P-tau181 ratio showed potential diagnostic and progression monitoring value, respectively, for Alzheimer’s disease patients.

## Teaser

Sun *et al*. analysed plasma biomarkers in a Chinese cohort to diagnose Alzheimer’s disease and distinguish it from other dementias. Phosphorylated tau protein at threonine 181 effectively identified patients with Alzheimer’s disease, while the amyloid-beta 42 to phosphorylated tau 181 ratio showed potential for tracking disease progression.

## Graphical Abstract

**Graphical Abstract:**
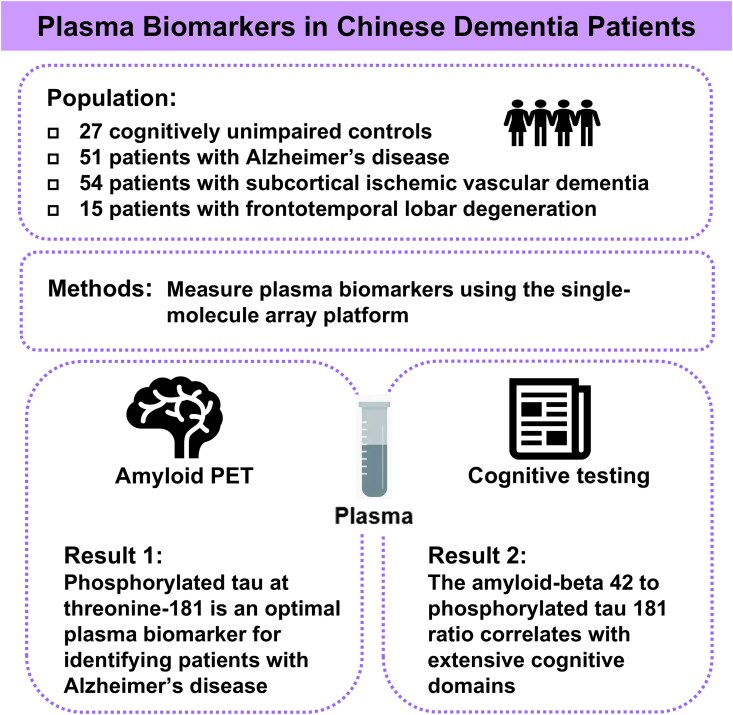

## Introduction

Biomarkers, substances that reflect pathological or pathophysiological processes, have been widely investigated for neurodegenerative diseases in the past decade and incorporated or recommended in various diagnostic criteria, such as those of the International Working Group (IWG)^1^ and the National Institute on Aging and Alzheimer’s Association (NIA-AA) Research Framework for Alzheimer’s disease.^2^ Currently, biomarkers are mainly measured in the CSF or by PET. Given the invasiveness of obtaining CSF and the high cost and limited access to PET, identifying and analysing more readily available biomarkers are necessary for clinical practice. Blood-based biomarkers are ideally suited for use at the population level for screening, diagnosis and tracking disease progression, and their sensitivity has been largely improved by ultrasensitive assays, such as the single-molecule array (Simoa).^3,4^

The plasma levels of biomarkers such as amyloid β (Aβ) 42, Aβ40, total tau (T-tau), phosphorylated tau (P-tau) and neurofilament protein light chain (NfL) were demonstrated to be correlated with their levels in the CSF^5-8^ and Aβ and tau deposition on PET.^9-11^ Several studies have reported the performance of plasma biomarkers in identifying patients with Alzheimer’s disease and differentiating this disease from other types of dementia, such as frontotemporal lobar degeneration (FTLD) spectrum and Lewy body dementia (LBD).^12-14^ In addition to the detection of core pathology as suggested by the AT(N) system, glial fibrillar acidic protein (GFAP), a marker of astrocytic activation and neuroinflammation, has also been observed to be elevated in the plasma of patients with early Alzheimer’s disease and individuals at risk of Alzheimer’s disease^15,16^ and is associated with Alzheimer’s disease pathology and Alzheimer’s disease dementia conversion in patients with mild cognitive impairment (MCI).^17^

Most previous findings were based on predominantly Caucasian populations from Western countries. A few studies have recently explored blood biomarkers for the screening, diagnosis and prediction of Alzheimer’s disease in the Chinese population.^18-20^ However, the value of plasma biomarkers in differentiating Alzheimer’s disease from other common types of dementia, such as vascular dementia (VaD), particularly in East Asia, is still inconclusive.

Recently, reference intervals for plasma NfL, Aβ42, Aβ40, T-tau and P-tau181 measured with ultrasensitive Simoa in a Chinese healthy population were established in our laboratory.^20,21^ The aim of the present study was to compare the profiles of plasma biomarkers (Aβ42, Aβ40, T-tau, P-tau181, NfL and GFAP) in a Chinese population including cognitively unimpaired (CU) individuals and patients with Alzheimer’s disease, subcortical ischaemic vascular dementia (SIVD) and FTLD, whose diagnoses were supported by amyloid PET or multimodal MRI scan. Their diagnostic and differentiating abilities in patients with different types of dementia were further identified. Furthermore, the correlation between plasma biomarkers and domain-specific cognitive functions was analysed in Alzheimer’s disease patients.

## Materials and methods

### Participants

This study was approved by the Ethics Committee of Tianjin Medical University General Hospital. Written informed consent was obtained from all participants. A total of 147 participants, including 51 patients with Alzheimer’s disease, 54 patients with SIVD, 15 patients with FTLD [seven with the behavioural variant of frontotemporal dementia (bvFTD), three with nonfluent/agrammatic variant primary progressive aphasia (nfvPPA) and five with semantic variant primary progressive aphasia (svPPA)] and 27 CU controls, were recruited from the memory clinic of Tianjin Medical University General Hospital from June 2017 to September 2020. All participants were aged 50–85 years, had an educational level of no <3 years, and underwent a comprehensive evaluation, including medical history, physical examination, clinical laboratory tests, brain MRI and neuropsychological assessments. Alzheimer’s disease patients had to meet the diagnostic criteria of the IWG-2,^22^ presenting with early and prominent episodic memory impairment and a positive result on ^11^C-labeled Pittsburgh compound B (PiB) PET. SIVD patients had to meet the Vascular Behavioural and Cognitive Disorders diagnostic criteria for VaD^23^ and show evidence of subcortical ischaemic lesions on brain MRI, such as white matter hyperintensities, lacunes, enlarged perivascular spaces and microbleeds, without large cortical infarction. Patients with FTLD were diagnosed based on the criteria for bvFTD,^24^ nfvPPA and svPPA.^25^ All patients had a Mini-Mental Status Examination (MMSE) score within the range of 10–26 and a Clinical Dementia Rating (CDR) score within 0.5–2. Patients whose cognitive decline was caused by other neurological diseases, mental disorders, or medical conditions, such as LBD, normal pressure hydrocephalus, multiple sclerosis, severe depression, vitamin B12 deficiency and thyroid dysfunction, were excluded. CU controls had no cognitive complaints or objective cognitive impairment, could independently perform activities of daily living, and had a CDR score = 0 and an MMSE score > 26.

### PET image acquisition and interpretation

PiB PET scanning was conducted at the PET/CT centre of Tianjin Medical University General Hospital on a Discovery PET/CT 710 scanner (GE Healthcare) in the 3D scanning mode according to the procedure described in our previous studies.^26^ PiB was injected into an antecubital vein as a bolus, with a mean dose of 370–555 MBq. Images were acquired during a 90 min dynamic PET scan (34 frames: 4 × 15 s, 8 × 30 s, 9 × 60 s, 2 × 180 s, 8 × 300 s, 3 × 600 s). Each frame produced 47 slices 3.75 mm thick, which covered the whole brain. PiB images were reconstructed into a 256 × 256 matrix (pixel size of 1.37 mm^2^).

The positivity or negativity of PiB PET was determined by the ratio of the mean value of the target region to that of the cerebellum, with a cutoff value of 1.5 (the upper 95% confidence interval from a cluster analysis of healthy individuals).

### Neuropsychological assessments

In addition to the MMSE and Montreal Cognitive Assessment (MoCA) for global cognition, a neuropsychological battery was performed to evaluate various cognitive domains,^27^ including the Rey Auditory Verbal Learning Test (AVLT) for memory, the Animal Verbal Fluency Test (AFT), the Controlled Oral Word Association Test (COWAT) and the Boston Naming Test (BNT) for language, the Symbol Digit Modalities Test (SDMT) and the Trail Making Test-A (TMT-A) for attention and information processing speed, the Trail Making Test-B (TMT-B) and the Stroop colour-word test for executive function, and the Benton Judgment of Line Orientation (JLO) for visuospatial function. TMT-A and TMT-B scores were multiplied by −1 so that for all tests, a higher score indicated better performance on the specific tasks. Raw scores were converted to *Z*-scores for the five cognitive domains using the mean and standard deviation (SD) of the CU group.

### Blood sample collection and processing

Blood samples were obtained by venipuncture, collected in EDTA tubes, centrifuged to isolate plasma, aliquoted and stored at −80°C until further analyses. Plasma biomarkers were measured using Simoa technology with an ultrahigh sensitivity protein molecular detection instrument (Simoa HD-1, Quanterix, MA, USA) according to the manufacturer’s protocol (Quanterix Corp, Billerica, MA, USA). Plasma Aβ42, Aβ40 and T-tau were measured with a Neurology 3-Plex A assay kit in the Alzheimer’s disease and CU groups. Plasma GFAP and NfL were measured with a Neurology 2-Plex B assay kit, and plasma P-tau181 was measured with an Advantage V2 assay in all groups. The lower limits of detection of the Aβ42, Aβ40, T-tau, P-taul8l, NfL and GFAP assays were 0.045, 0.196, 0.019, 0.028, 0.065 and 0.475 pg/mL, whereas the lower levels of quantification (LLOQ) were 0.142, 0.675, 0.063, 0.338, 0.200 and 4.15 pg/mL, respectively. Samples were diluted four times and thoroughly mixed with magnetic beads to form magnetic bead immune complexes with fluorescence. Simoa used a butterfly chip to capture magnetic bead immune complexes and ultrasensitive fluorescent probes to detect fluorescent signals. All samples were measured in duplicate, and the mean of the duplicate values was taken as the final readout. Two quality control samples were run on each plate for each analyte. All samples were analysed simultaneously using the same batch of reagents. All samples were measured well above the kit LLOQ. Plasma P-tau181 was not available for one participant in the Alzheimer’s disease group and five participants in the FTLD group because of limited sample availability.

### Statistical analyses

Statistical Package for Social Science (SPSS version 25) and GraphPad Prism (version 9) were used for statistical analyses, and significance was set at *P* < 0.05. We assessed the normality of the data with the Kolmogorov–Smirnov test. For continuous variables, groups were compared using one-way analysis of variance [normally distributed data, including age and body mass index (BMI)] or Kruskal–Wallis tests (skewed data, including education and disease course). Categorical variables were analysed with the Pearson χ^2^ (e.g. sex) and Fisher’s exact tests (e.g. comorbidities). Cognitive test scores were compared using ANCOVA adjusted for age, sex and educational level. Plasma biomarkers were compared using analysis of covariance (ANCOVA) to test the main effect of the diagnostic group, with age and sex as covariates. Log-transformed data were used for some biomarkers (e.g. P-tau181, NfL and GFAP) that were not normally distributed. Post hoc pairwise comparisons were carried out using the Bonferroni correction method for ANCOVA. Correlations between demographic data and plasma biomarkers were analysed using Spearman rank correlations in CU controls.

Classification analyses were performed using receiver operating characteristic (ROC) curve analyses to estimate the diagnostic ability of the plasma biomarkers using MedCalc 19.1. The optimal cutoff scores were calculated based on the maximum sum of sensitivity and specificity obtained from the ROC curve, and the area under the curve (AUC), sensitivity and specificity were reported for each comparison across the four groups.

The cognitive correlation of each plasma biomarker was analysed using Spearman rank correlations for each diagnostic group. Scatter plots were used to illustrate the correlations.

## Results

### Demographics and clinical characteristics of all participants

Demographics, clinical information and cognitive scores across the different diagnostic groups are shown in Table 1. There were significant differences in age (67.61 ± 7.48 versus 70.63 ± 6.19 versus 62.53 ± 7.84 versus 67.04 ± 6.51, years), sex (female, 68.6% versus 33.3% versus 60.0% versus 48.1%) and BMI (22.62 ± 2.54 versus 24.39 ± 3.12 versus 23.55 ± 2.30 versus 24.95 ± 2.66, kg/m^2^) among the Alzheimer’s disease, SIVD, FTLD and CU groups. Specifically, patients with FTLD were significantly younger than SIVD patients (*P* < 0.001). The Alzheimer’s disease group had a higher proportion of females than the CU (*P* < 0.05) and SIVD (*P* < 0.05) groups; the SIVD group had a lower proportion of females than the FTLD group (*P* < 0.05). Alzheimer’s disease patients had a lower BMI than CU controls and SIVD patients (*P* < 0.05). There was no significant difference in education (*P* = 0.366) or disease course among patient groups (*P* = 0.074). In terms of comorbidities, the Alzheimer’s disease group had a lower proportion of hypertension than the SIVD, FTLD and CU groups (17.6% versus 75.9% versus 26.7% versus 48.1%, *P* < 0.05). The SIVD group had a higher proportion of diabetes than the Alzheimer’s disease and CU groups (27.8% versus 7.8% versus 7.4%, *P* < 0.05) and had a higher proportion of stroke than the Alzheimer’s disease, FTLD and CU groups (37.0% versus 2.0% versus 0% versus 0%, *P* < 0.05). There were no significant differences in the proportions of dyslipidemia and myocardial infarction among the groups.

**Table 1:** Summary of participant characteristics and cognitive performance per diagnostic group

	Alzheimer’s disease	SIVD	FTLD	CU	*P*
Maximum, *n*	51	54	15	27	
Age, years	67.61 (7.48)	70.63 (6.19)	62.53 (7.84)^c^	67.04 (6.51)	0.001
Female, *n* (%)	35 (68.6)^a^	18 (33.3)^b^	9 (60.0)^c^	13 (48.1)	0.003
Education, years	10.69 (3.32)	10.96 (3.19)	11.40 (2.75)	12.00 (3.27)	0.366
Disease course, years	3.12 (2.47)	2.64 (2.02)	4.20 (2.48)^c^	NA	0.074
BMI, kg/m^2^	22.62 (2.54)^a^	24.39 (3.12)^b^	23.55 (2.30)	24.95 (2.66)	0.002
Comorbidities					
Hypertension, *n* (%)	9 (17.6)^a^	41 (75.9)^a,b^	4 (26.7)^b,c^	13 (48.1)	<0.001
Dyslipidemia, *n* (%)	4 (7.8)	3 (5.6)	0 (0)	2 (7.4)	0.718
Diabetes, *n* (%)	4 (7.8)	15 (27.8)^a,b^	1 (6.7)	2 (7.4)	0.012
Stroke, *n* (%)	1 (2.0)	20 (37.0)^a,b^	0 (0)^c^	0 (0)	<0.001
Myocardial infarction, *n* (%)	1 (2.0)	2 (3.7)	0 (0)	1 (3.7)	0.843
Cognitive score					
MMSE	18.41 (5.33)^a^	21.87 (4.59)^a,b^	17.73 (6.64)^a^	27.56 (1.42)	<0.001
MoCA	14.05 (5.13)^a^	16.79 (5.35)^a^	12.40 (5.90)^a^	24.88 (3.08)	<0.001
Memory	−2.83 (0.64)^a^	−2.17 (0.87)^a^	−2.38 (0.95)^a^	0.028 (0.88)	<0.001
Language	−1.95 (2.27)^a^	−1.45 (1.38)^a^	−5.49 (4.61)^a,b,c^	−0.011 (0.61)	<0.001
Information processing speed	−1.95 (1.26)^a^	−1.60 (1.13)^a^	−1.43 (1.17)^a^	0.002 (0.91)	<0.001
Executive function	−1.92 (1.04)^a^	−1.63 (0.95)^a^	−1.61 (1.10)^a^	0.038 (0.83)	<0.001
Visuospatial function	−2.41 (2.21)^a^	−1.25 (1.53)^a^	−1.06 (1.61)	0.001 (1.00)	<0.001

Data are presented as the means (SD) unless otherwise specified.

One-way analyses of variance and Kruskal–Wallis tests were used to compare continuous variables, and Pearson *χ*^2^ and Fisher’s exact tests were used to compare categorical variables. Cognitive test scores were assessed by ANCOVA adjusting for age, sex and education. The MMSE and MoCA results are presented as raw scores, and cognitive domains are presented as *Z*-scores.

CU, cognitively unimpaired; SIVD, subcortical ischaemic vascular dementia; FTLD, frontotemporal lobar degeneration; BMI, body mass index; MMSE, Mini-Mental State Examination; MoCA, Montreal Cognitive Assessment Scale; NA, not applicable.

^a^ *P* < 0.05 versus CU.

^b^ *P* < 0.05 versus Alzheimer’s disease.

^c^ *P* < 0.05 versus SIVD.

The MMSE score (18.41 ± 5.33 versus 21.87 ± 4.59 versus 17.73 ± 6.64 versus 27.56 ± 1.42) and MoCA score (14.05 ± 5.13 versus 16.79 ± 5.35 versus 12.40 ± 5.90 versus 24.88 ± 3.08) were significantly lower in the Alzheimer’s disease, SIVD and FTLD groups, respectively, than the CU group (*P* < 0.001), and the MMSE score was lower in patients with Alzheimer’s disease than in patients with SIVD (*P* < 0.05). *Z*-scores for almost all cognitive domains, including memory, language, information processing speed, executive function and visuospatial function, were prominently decreased in the three patient groups relative to the CU group; however, there was no significant difference in visuospatial function between the FTLD group and the CU group.

### Plasma biomarker levels between different groups

**Figure 1:**
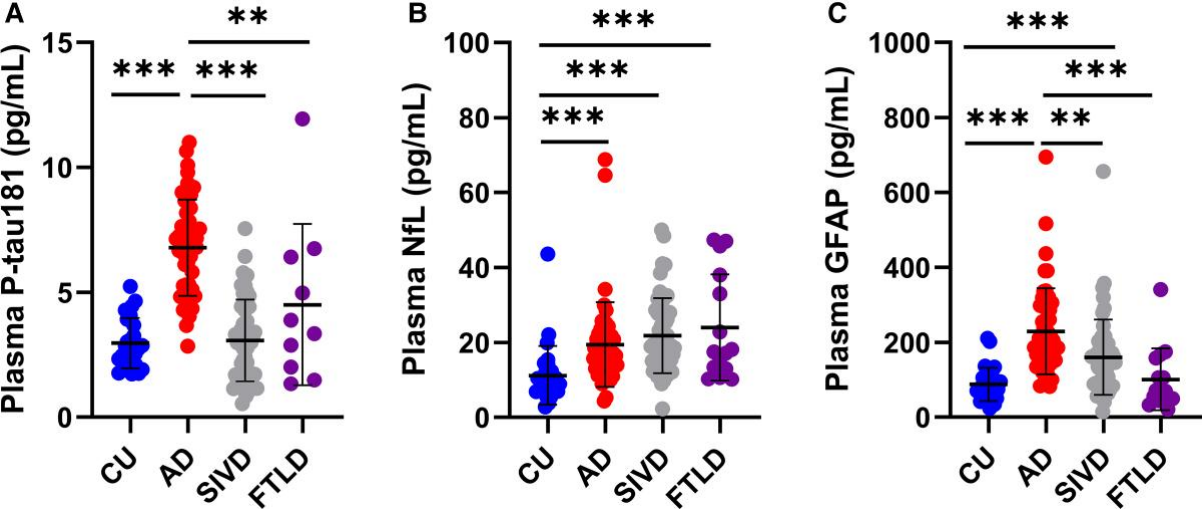
**Comparisons of plasma levels of P-tau181, NfL and GFAP among the groups.** (**A**) P-tau181 was greater in the Alzheimer’s disease group than in the SIVD, FTLD and CU groups. (**B**) NfL was greater in all patient groups than in the CU group. (**C**) GFAP was greater in the Alzheimer’s disease group than in all other groups and greater in the SIVD group than in the CU group. Points represent the value of the plasma marker in each diagnostic group. Comparisons were performed using the log-transformed values with ANCOVA after adjusting for age and sex. CU, *n* = 27; Alzheimer’s disease, *n* = 51 (P-tau181 assay, *n* = 50); SIVD, *n* = 54; FTLD, *n* = 15 (P-tau181 assay, *n* = 10). ***P < 0.001, **P < 0.01. CU, cognitively unimpaired; SIVD, subcortical ischaemic vascular dementia; FTLD, frontotemporal lobar degeneration; P-tau181, phosphorylated tau181; NfL, neurofilament light chain; GFAP, glial fibrillary acidic protein.

Plasma levels of P-tau181, NfL and GFAP differed among the four groups after adjusting for age and sex (Table 2, Fig. 1). The P-tau181 level was greater in the Alzheimer’s disease group (6.79 ± 1.92 pg/mL) than in the CU group (2.98 ± 1.01 pg/mL, *P* < 0.001) and the other two patient groups (SIVD, 3.08 ± 1.63 pg/mL, *P* < 0.001; FTLD, 4.51 ± 3.23 pg/mL, *P* < 0.01). Plasma NfL was greater in the Alzheimer’s disease group (19.44 ± 11.36 pg/mL, *P* < 0.001), the SIVD group (21.85 ± 10.00 pg/mL, *P* < 0.001) and the FTLD group (24.01 ± 14.17 pg/mL, *P* < 0.001) than in the CU group (11.23 ± 7.85 pg/mL). The plasma GFAP level was greater in the Alzheimer’s disease group (229.46 ± 114.47 pg/mL) than in the CU group (87.92 ± 44.84 pg/mL, *P* < 0.001) and the other two patient groups (SIVD, 160.14 ± 100.94 pg/mL, *P* < 0.01; FTLD, 101.52 ± 82.53 pg/mL, *P* < 0.001) and was greater in the SIVD group than in the CU group (*P* < 0.001) but did not significantly differ between the FTLD and CU groups (*P* > 0.05).

**Table 2:** Plasma biomarker concentrations per diagnostic group

	Alzheimer’s disease (*N* = 51)	SIVD (*N* = 54)	FTLD (*N* = 15)	CU (*N* = 27)	*P*
Aβ42, pg/mL	13.74 (4.92)			17.16 (4.37)	0.004
Aβ40, pg/mL	204.97 (44.4)			229.77 (50.3)	0.042
T-tau, pg/mL	2.80 (1.07)			2.32 (0.77)	0.045
P-tau181, pg/mL	6.79 (1.92)^a^	3.08 (1.63)^b^	4.51 (3.23)^b^	2.98 (1.01)	<0.001
NfL, pg/mL	19.44 (11.36)^a^	21.85 (10.00)^a^	24.01 (14.17)^a^	11.23 (7.85)	<0.001
GFAP, pg/mL	229.46 (114.47)^a^	160.14 (100.94)^a,b^	101.52 (82.53)^b^	87.92 (44.84)	<0.001

Data are presented as the means (SD). Comparisons were performed using ANCOVA adjusting for age and sex. The plasma P-tau181, NfL and GFAP levels were not normally distributed, and log-transformed data were used. Plasma P-tau181 was not available for one participant in the Alzheimer’s disease group and five participants in the FTLD group.

CU, cognitively unimpaired; SIVD, subcortical ischaemic vascular dementia; FTLD, frontotemporal lobar degeneration; Aβ, β-amyloid; T-tau, total tau; P-tau181, phosphorylated tau181; NfL, neurofilament light chain; GFAP, glial fibrillary acidic protein.

^a^ *P* < 0.01 versus CU.

^b^ *P* < 0.01 versus Alzheimer’s disease.

**Figure 2:**
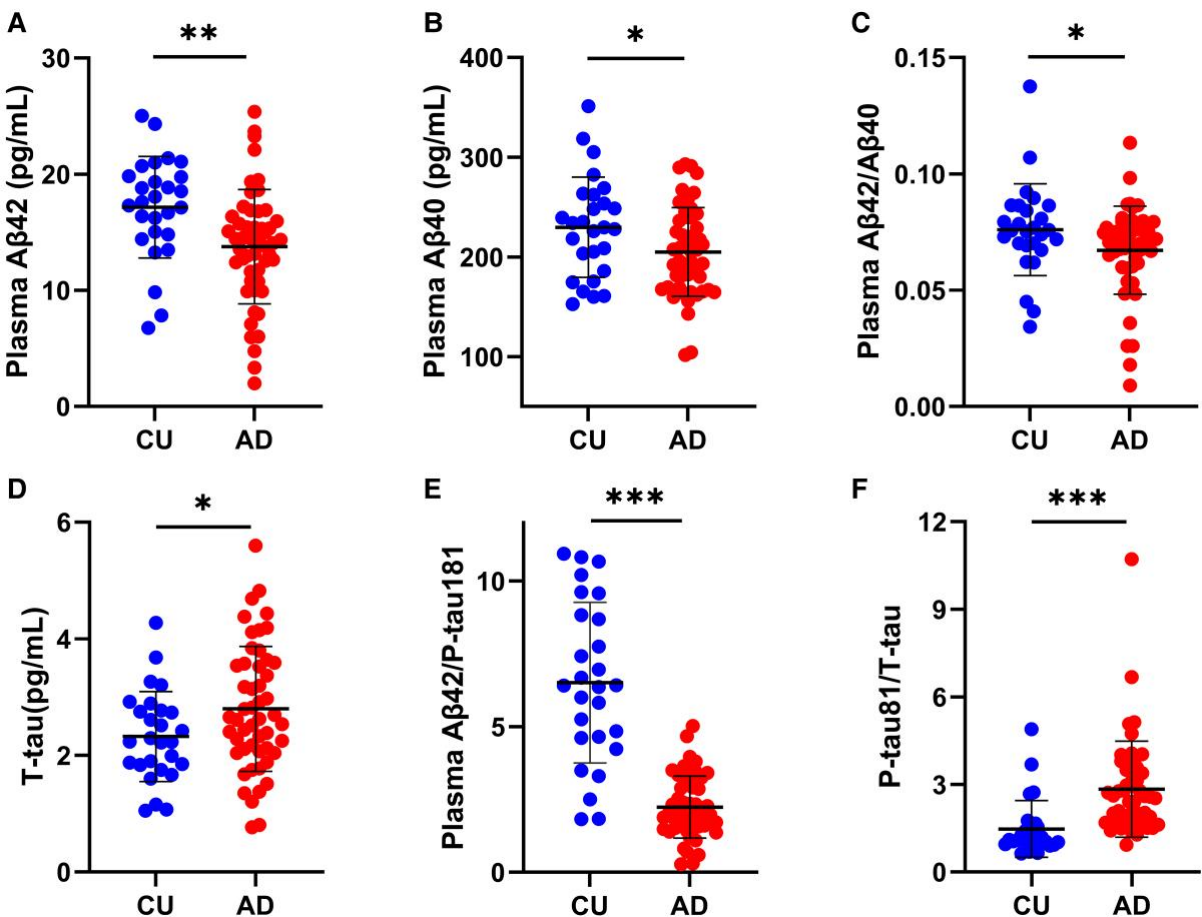
**Comparisons of plasma levels of Aβ and tau between Alzheimer’s disease patients and CU controls.** Plasma levels of Aβ42 (**A**) and Aβ40 (**B**) and plasma Aβ42/Aβ40 (**C**) and Aβ42/P-tau181 (**E**) were lower, and plasma levels of T-tau (**D**) and P-tau181/T-tau (**F**) were higher in the Alzheimer’s disease group than in the CU group. Points represent the value of the plasma marker in the CU and Alzheimer’s disease groups. Comparisons were performed using ANCOVA adjusting for age and sex. The P-tau181/T-tau were not normally distributed, and log-transformed data were used. CU, *n* = 27; Alzheimer’s disease, *n* = 51 (P-tau181 assay, *n* = 50). ***P < 0.001, **P < 0.01, *P < 0.05. CU, cognitively unimpaired; Alzheimer’s disease, Alzheimer’s disease; Aβ, β-amyloid; T-tau, total tau; P-tau181, phosphorylated tau181.

Plasma Aβ42, Aβ40 and T-tau were only tested in patients with Alzheimer’s disease and CU controls (Table 2 and Fig. 2). Alzheimer’s disease patients had lower plasma levels of Aβ42 (13.74 ± 4.92 versus 17.16 ± 4.37 pg/mL, *P* = 0.004) and Aβ40 (204.97 ± 44.4 versus 229.77 ± 50.3 pg/mL, *P* = 0.042) and higher plasma levels of T-tau (2.80 ± 1.07 versus 2.32 ± 0.77 pg/mL, *P* = 0.045) than CU controls. Moreover, the Aβ42/Aβ40 (0.067 ± 0.019 versus 0.076 ± 0.020, *P* = 0.042) and Aβ42/P-tau181 ratios (2.24 ± 1.07 versus 6.51 ± 2.76, *P* < 0.001) were significantly lower, and the P-tau 181/T-tau ratio (2.84 ± 1.65 versus 1.48 ± 0.97, *P* < 0.001) was significantly higher, in Alzheimer’s disease patients than in CU controls.

In terms of associations between biomarkers and demographic characteristics in the CU group, only plasma NfL showed a positive correlation with age (*r* = 0.511, *P* = 0.007).

### Diagnostic and differential performances of plasma biomarkers

**Figure 3:**
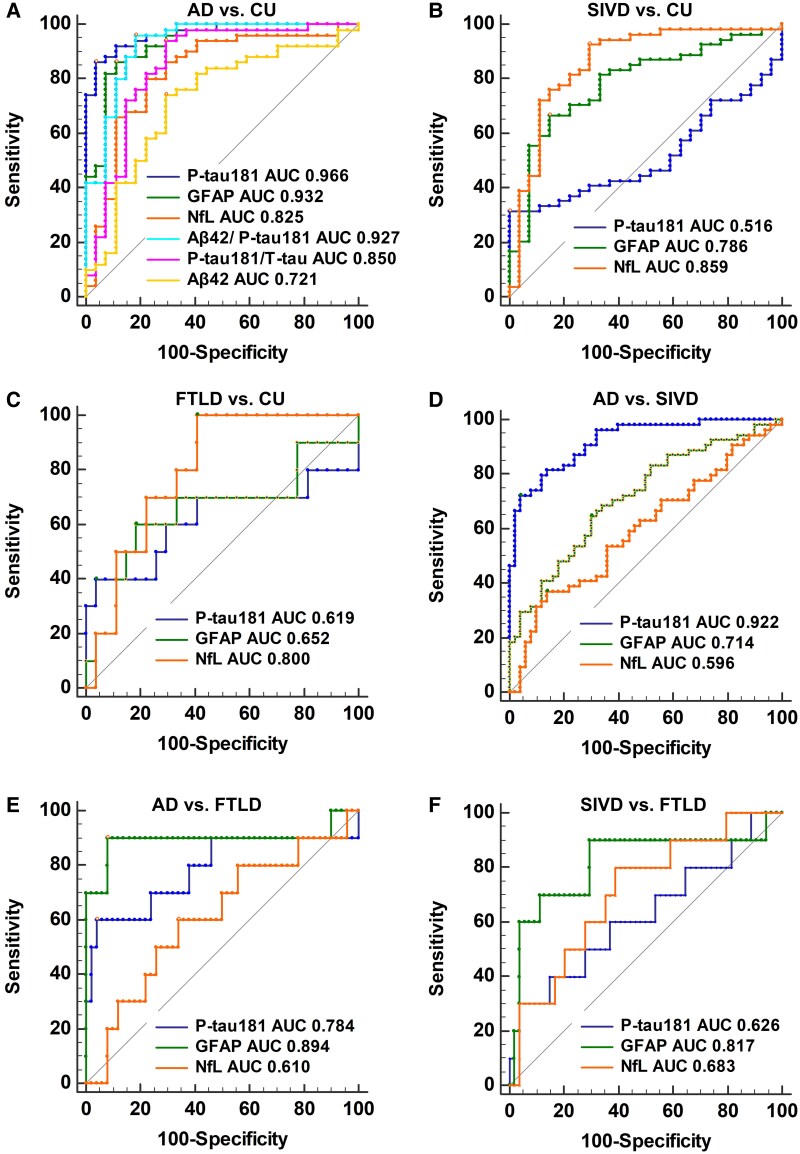
**ROC curve analysis for the plasma biomarkers in the diagnosis and differential diagnosis of various types of dementia.** (**A**) In discriminating Alzheimer’s disease patients from CU controls, plasma P-tau181 showed the highest AUC, followed by GFAP, Aβ42/P-tau181, P-tau181/T-tau, NfL and Aβ42. (**B**) In discriminating SIVD patients from CU controls, plasma NfL showed the highest AUC, followed by GFAP. (**C**) In discriminating patients with FTLD from CU controls, only plasma NfL showed a satisfactory AUC, while plasma P-tau181 and GFAP showed lower diagnostic values. (**D**) In distinguishing patients with Alzheimer’s disease from patients with SIVD, plasma P-tau181 showed the highest AUC, followed by GFAP. (**E**) Plasma GFAP showed a better performance than P-tau181 in distinguishing Alzheimer’s disease patients from FTLD patients. (**F**) Plasma GFAP also showed good performance in discriminating SIVD patients from FTLD patients, followed by NfL and P-tau181. CU, *n* = 27; Alzheimer’s disease, *n* = 51 (P-tau181 assay, *n* = 50); SIVD, *n* = 54; FTLD, *n* = 15 (P-tau181 assay, *n* = 10).

The diagnostic and differential performances of plasma biomarkers for patients with Alzheimer’s disease, SIVD and FTLD are presented in Table 3 and Fig. 3. In discriminating Alzheimer’s disease patients from CU controls (Fig. 3A), P-tau181 showed the highest AUC of 0.966, with a sensitivity of 0.86 and a specificity of 0.96 at a cutoff value of 4.65 pg/mL, followed by GFAP (AUC 0.932, sensitivity 0.86, specificity 0.89), Aβ42/P-tau181 (AUC 0.927, sensitivity 0.96, specificity 0.82), P-tau181/T-tau (AUC 0.850, sensitivity 0.94, specificity 0.70), NfL (AUC 0.825, sensitivity 0.80, specificity 0.78), Aβ42 (AUC 0.721, sensitivity 0.75, specificity 0.70), T-tau (AUC 0.630, sensitivity 0.39, specificity 0.85), Aβ40 (AUC 0.629, sensitivity 0.73, specificity 0.59) and Aβ42/Aβ40 (AUC 0.627, sensitivity 0.71, specificity 0.56).

**Table 3:** Diagnostic performance of plasma biomarkers

	Plasma biomarker	AUC	Cutoff (pg/mL)	Sensitivity	Specificity
Distinguishing Alzheimer’s disease from CU					
	P-tau181	0.966	4.65	0.86	0.96
	GFAP	0.932	134.14	0.86	0.89
	NfL	0.825	12.37	0.80	0.78
	Aβ42	0.721	15.95	0.75	0.70
	Aβ40	0.629	225.245	0.71	0.59
	Aβ42/Aβ40	0.627	0.075	0.73	0.56
	Aβ42/P-tau181	0.927	3.96	0.96	0.82
	T-tau	0.630	2.918	0.39	0.85
	P-tau181/T-tau	0.850	1.416	0.94	0.70
Distinguishing SIVD from CU					
	P-tau181	0.516	1.73	0.32	1.00
	GFAP	0.786	124.77	0.67	0.85
	NfL	0.859	11.19	0.93	0.70
Distinguishing FTLD from CU					
	P-tau181	0.619	4.65	0.40	0.96
	GFAP	0.652	55.21	0.60	0.82
	NfL	0.800	9.89	1.00	0.59
Distinguishing Alzheimer’s disease from SIVD					
	P-tau181	0.922	3.86	0.72	0.96
	GFAP	0.714	163.80	0.65	0.71
	NfL	0.596	24.46	0.37	0.86
Distinguishing Alzheimer’s disease from FTLD					
	P-tau181	0.784	3.89	0.60	0.96
	GFAP	0.894	106.44	0.90	0.92
	NfL	0.610	15.60	0.60	0.67
Distinguishing SIVD from FTLD					
	P-tau181	0.626	5.79	0.30	0.96
	GFAP	0.817	71.18	0.90	0.70
	NfL	0.683	18.20	0.72	0.59

AUC, area under the curve; CU, cognitively unimpaired; SIVD, subcortical ischaemic vascular dementia; FTLD, frontotemporal lobar degeneration; Aβ, β-amyloid; P-tau181, phosphorylated tau181; NfL, neurofilament light chain; GFAP, glial fibrillary acidic protein.

Plasma NfL showed high AUCs of 0.859 (sensitivity 0.93, specificity 0.70, at a cutoff value of 11.19 pg/mL) and 0.800 (sensitivity 1.00, specificity 0.59, at a cutoff value of 9.89 pg/mL) in distinguishing patients with SIVD (Fig. 3B) and patients with FTLD (Fig. 3C) from CU controls, respectively. GFAP also showed a satisfactory AUC of 0.786 (sensitivity 0.67, specificity 0.85, at a cutoff value of 124.77 pg/mL) in distinguishing patients with SIVD from CU controls (Fig. 3B).

In distinguishing patients with Alzheimer’s disease from patients with SIVD (Fig. 3D), plasma P-tau181 showed an AUC of 0.922 with a sensitivity of 0.72 and a specificity of 0.96 at a cutoff value of 3.86 pg/mL, followed by GFAP (AUC 0.714, sensitivity 0.65, specificity 0.71). Plasma GFAP showed a better performance (AUC 0.894, sensitivity 0.90, specificity 0.92, at a cutoff value of 106.44 pg/mL) in distinguishing Alzheimer’s disease patients from FTLD patients (Fig. 3E) than P-tau181 (AUC 0.784, sensitivity 0.60, specificity 0.96, at a cutoff value of 3.89 pg/mL) and a better performance (AUC 0.817, sensitivity 0.90, specificity 0.70, at a cutoff value of 71.18 pg/mL) in discriminating SIVD patients from FTLD patients (Fig. 3F) than NfL (AUC 0.683, sensitivity 0.72, specificity 0.59) and P-tau181 (AUC 0.626, sensitivity 0.30, specificity 0.96).

### Correlations between cognitive test scores and plasma biomarkers

**Figure 4:**
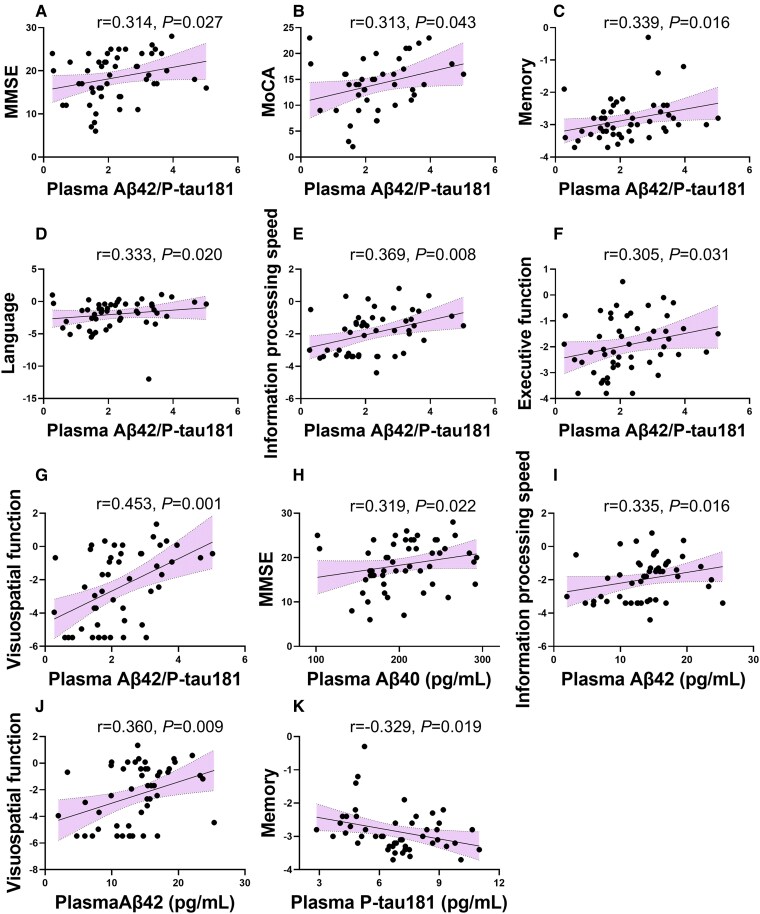
**Correlations between plasma biomarkers and cognitive function in Alzheimer’s disease patients.** Aβ42/P-tau181 showed positive correlations with the MMSE (**A**) and MoCA scores (**B**) and the Z scores of various cognitive domains, including memory (**C**), language (**D**), attention and information processing speed (**E**), executive function (**F**) and visuospatial function (**G**). Aβ40 was positively correlated with the MMSE score (**H**). Aβ42 was positively correlated with information processing speed (**I**) and visuospatial function (**J**). P-tau181 was negatively correlated with memory (**K**). Correlations were analysed using Spearman rank correlations. (**A–G**) and (**K**), *n* = 50; (**H–J**), *n* = 51.

After controlling for age, sex and education, the plasma biomarkers of core pathology were correlated with cognitive function in Alzheimer’s disease patients (Fig. 4). The Aβ42/P-tau181 ratio showed extensively positive correlations with cognitive scores, including the MMSE score (*r* = 0.314, *P* = 0.027), MoCA (*r* = 0.313, *P* = 0.043) and *Z*-scores of various cognitive domains, such as memory (*r* = 0.339, *P* = 0.016), language (*r* = 0.333, *P* = 0.020), attention and information processing speed (*r* = 0.369, *P* = 0.008), executive function (*r* = 0.305, *P* = 0.031) and visuospatial function (*r* = 0.453, *P* = 0.001). In addition, Aβ42 was positively correlated with information processing speed (*r* = 0.335, *P* = 0.016) and visuospatial function (*r* = 0.360, *P* = 0.009), Aβ40 was positively correlated with the MMSE score (*r* = 0.319, *P* = 0.022) and P-tau181 was negatively correlated with memory (*r* = −0.329, *P* = 0.019). No correlations between cognitive function and plasma T-tau, NfL and GFAP were observed in Alzheimer’s disease patients.

The cognitive correlation of each biomarker in CU controls and patients with other types of dementia was also analysed. The results revealed that plasma NfL was negatively correlated with the MMSE (*r* = −0.604, *P* = 0.017) and MoCA (*r* = −0.594, *P* = 0.020) scores in the FTLD group, but no other significant associations were observed between plasma biomarkers or other cognitive domains in the CU group, SIVD group or FTLD group.

## Discussion

In this study, we observed changes in plasma biomarkers in a Chinese cohort presenting with various types of dementia. Plasma P-tau181 had excellent diagnostic value, outperforming other single plasma biomarkers in distinguishing Alzheimer’s disease patients from CU volunteers and effectively distinguished Alzheimer’s disease patients from those with other types of dementia, such as SIVD and FTLD. Interestingly, plasma GFAP was also prominently elevated in Alzheimer’s disease patients and moderately increased in SIVD patients but not in FTLD patients in this cohort. Furthermore, the ratio of Aβ42/P-tau181 showed more extensive correlations with cognitive functions in Alzheimer’s disease patients than other single biomarkers, suggesting its potential role in monitoring disease progression.

The plasma Aβ42 concentration and Aβ42/Aβ40 ratio only showed moderate to low diagnostic value for Alzheimer’s disease in this study. This result is in line with previous findings suggesting that the plasma Aβ concentration is not sufficiently sensitive to pathological changes in the brain,^18,19,28^ which is likely due to the influence of Aβ metabolism outside the central nervous system.^29,30^ Previous work has shown that the plasma Aβ42/Aβ40 ratio was only decreased by 10–20% in individuals with cerebral Aβ pathology, in contrast to a 40–60% decrease for CSF Aβ42/Aβ40.^8,10,31^ Similarly, no significant diagnostic value was found for plasma T-tau from our results, consistent with previous demonstrations.^28,32^

Threonine 181 is one site that is subject to tau hyperphosphorylation, and the resulting P-tau181 directly contributes to neurofibrillary tangle formation in the brain.^33^ We demonstrated that P-tau181 outperformed the other single plasma biomarkers in identifying Alzheimer’s disease patients whose diagnosis was confirmed by amyloid PET with an AUC of 0.996. This finding is consistent with previous studies based on both Western and Chinese populations, which showed a similar diagnostic accuracy between plasma P-tau181 and tau PET or CSF P-tau181.^12,14,19,34,35^ The present study further suggested that the excellent diagnostic value of P-tau181 was due mainly to an increased phosphorylation rate but not elevated T-tau levels because the P-tau181/T-tau ratio was still significantly greater in Alzheimer’s disease patients than in CU controls and presented good discriminative ability (AUC of 0.850), consistent with previous findings.^28^ In addition, although the performance was slightly worse (AUC 0.784) in the present study than in previous studies (AUC 0.80–0.96),^12,13,35^ our results supported a moderate performance of plasma P-tau181 in discriminating patients with Alzheimer’s disease from patients with FTLD spectrum.

Moreover, we found that plasma P-tau181 had excellent performance in differentiating Alzheimer’s disease patients from SIVD patients (AUC 0.922). A previous study showed that P-tau181 had high accuracy (AUC 0.868) in discriminating Aβ+ Alzheimer’s disease patients from Aβ− VaD patients.^36^ In this study, we only focused on SIVD, which is more difficult to differentiate from Alzheimer’s disease in clinical practice, because concomitant small vessel disease pathology is very common in elderly individuals, including Alzheimer’s disease patients,^19^ and SIVD patients usually present with a gradual progression that mimics Alzheimer’s disease. Our results suggest that plasma P-tau181 is a promising tool for distinguishing Alzheimer’s disease from SIVD.

Interestingly, higher plasma GFAP levels were also found in Alzheimer’s disease patients and SIVD patients, presenting excellent accuracy (AUC 0.932) and moderate performance (AUC 0.786) in identifying those patients, respectively. As a marker of astrocytic activation, GFAP reflects neuroinflammation and has been observed to be elevated in the blood in both persons with preclinical Alzheimer’s disease^15^ and Alzheimer’s disease patients (with both early and late onset)^16^ and showed an AUC of 0.95 in distinguishing Alzheimer’s disease patients from CUs in a recent study.^35^ However, this neuropathological process is not Alzheimer’s disease specific, and the plasma level of GFAP could also be increased after acute neuronal injury due to stroke or traumatic brain injury.^37^ Our results further suggested that GFAP levels could also be elevated in chronic cerebral ischaemia, as in patients with SIVD. We did not observe elevated plasma GFAP levels in FTLD patients, unlike some previous studies,^35,38^ probably due to the small sample size of the study or differences in the proportions of clinical and pathological phenotypes. However, consistent with most previous findings of a more significant increase in blood GFAP in Alzheimer’s disease patients than in FTLD patients,^12,13,38-40^ the plasma GFAP level was 2-fold higher in the Alzheimer’s disease group than in the FTLD group and effectively distinguished these two patient groups (AUC 0.894) in this study.

Although plasma NfL was greater in patients with various types of dementia than in CUs, there was no significant difference between Alzheimer’s disease, SIVD and FTLD patients. A few previous studies showed that FTLD patients had much higher levels of plasma NfL than Alzheimer’s disease patients.^12,14^ However, we did not find an effectively differentiating value of NfL for different types of dementia in this study. Since NfL was generally associated with neurodegeneration rather than a specific disease marker^41^ and its concentration was increased with natural aging,^21,42,43^ using NfL alone could not accurately distinguish dementia patients with different causes.

We found that the ratio of plasma Aβ42/P-tau181 had correlations with both global cognition and all cognitive domains in Alzheimer’s disease patients. The cognitive correlation of plasma P-tau181 has been observed in several previous studies^44-47^ and was demonstrated for memory ability in the present study, and the ratio of plasma Aβ42/P-tau181 has been found to correlate with MMSE score.^48^ Positive correlations between Aβ42 and information processing speed and visuospatial function and between Aβ40 and MMSE were also found in our study. Previous findings of correlations between cognition and single plasma amyloid biomarkers have been contradictory, possibly due to the diverse pathophysiological stages of Alzheimer’s disease patients.^45,46,49^ It has been suggested that the amyloid biomarkers of CSF and PET reflect the early pathological changes in Alzheimer’s disease but tend to reach a plateau in performance as the disease progressed even at the prodromal stage.^46^ In the present study, the combination of amyloid and P-tau181 (Aβ42/P-tau181) presented with more extensive cognitive correlations, indicating its better value in monitoring disease progression than any single biomarker.

There are several limitations to our study. We did not have postmortem confirmation of the diagnosis, nor could we compare the plasma findings with CSF or PET results for all participants. Therefore, our findings could be confounded by mixed pathologies or biological misdiagnosis, particularly in patients with SIVD who might have comorbid cerebrovascular disease pathology with other neurodegenerative diseases. Moreover, plasma P-tau217 was not measured in the present study, which has been found to more strongly correlate with Alzheimer’s disease neuropathology and have a better diagnostic ability compared with other P-tau biomarkers. Furthermore, it has been recommended in the revised criteria for diagnosis and staging of Alzheimer’s disease, in particular the %P-tau217 (ratio of P-tau217 to non-P-tau).^50^ In addition, Aβ42, Aβ40 and T-tau biomarkers were not assayed in the SIVD and FTLD groups. Their differential performances for Alzheimer’s disease from other types of dementia could not be determined in the present study. Finally, we had a small available number of samples from patients with FTLD, including different clinical phenotypes of bvFTD, nfPPA and svPPA. Moreover, since the pathology of FTLD is quite complex, biomarkers for discriminating different pathologies, such as TAR DNA binding protein and tau, are worthy of further investigation.

## Conclusion

Our results provide evidence from the Chinese population that blood biomarkers measured with ultrasensitive assays have potential value in Alzheimer’s disease diagnosis and differentiation, particularly P-tau181 for discriminating from SIVD and GFAP for discriminating from FTLD. Furthermore, the ratio of Aβ42/P-tau181 is a potential tool for Alzheimer’s disease progression monitoring, given its strong and more extensive cognitive correlations than any other individual biomarker.

## Data Availability

No new software and/or algorithms, in-house scripts or programmes were generated to support this study. All data generated or analysed during this study are included in this publication and/or are available from the corresponding author upon reasonable request.
